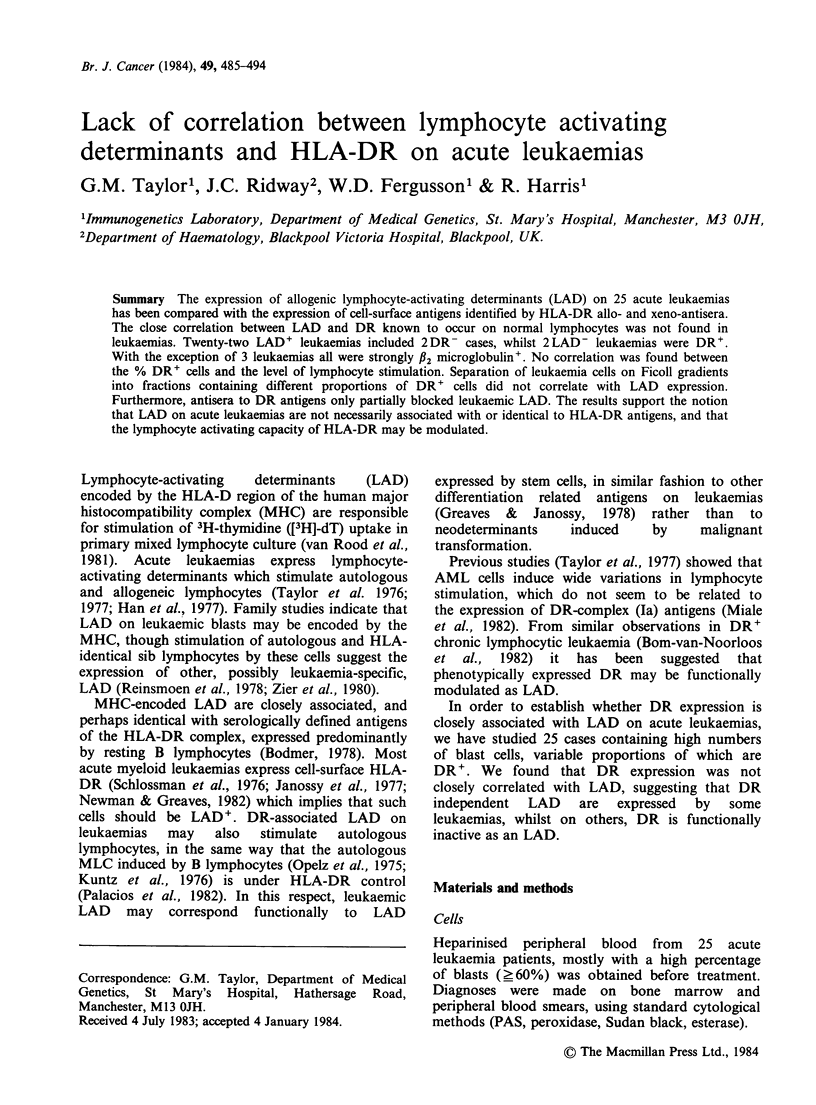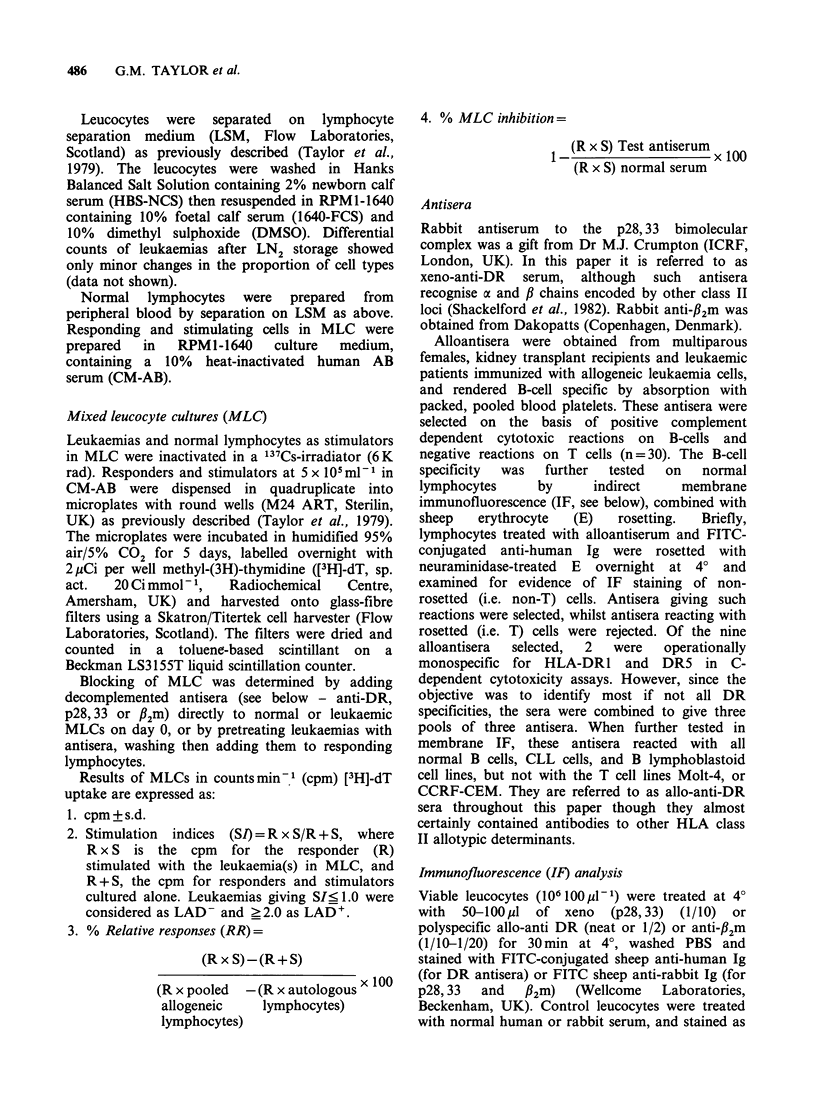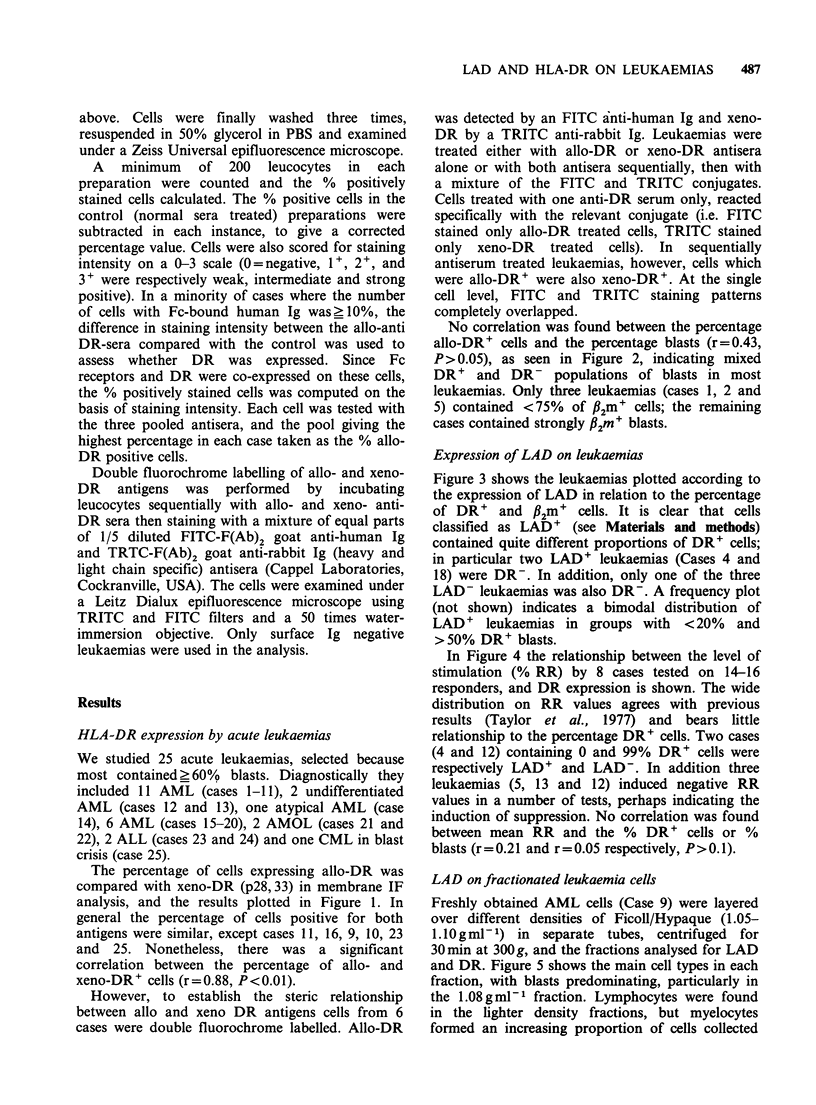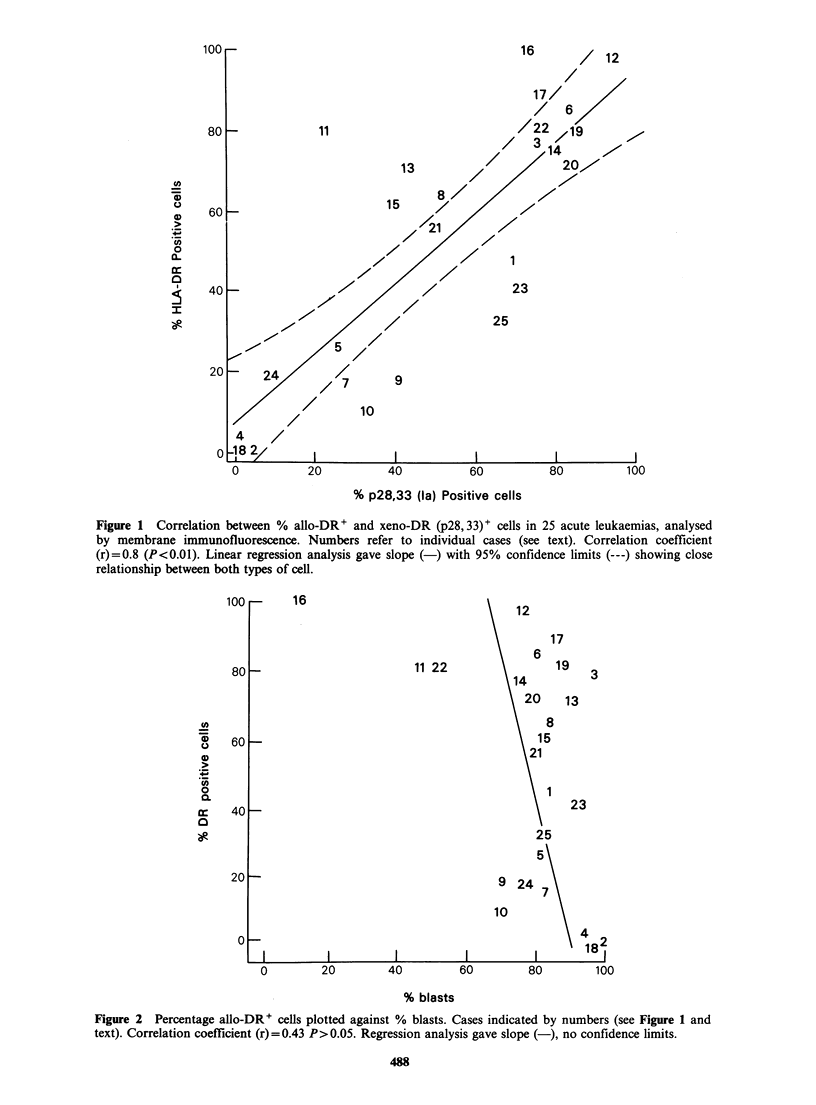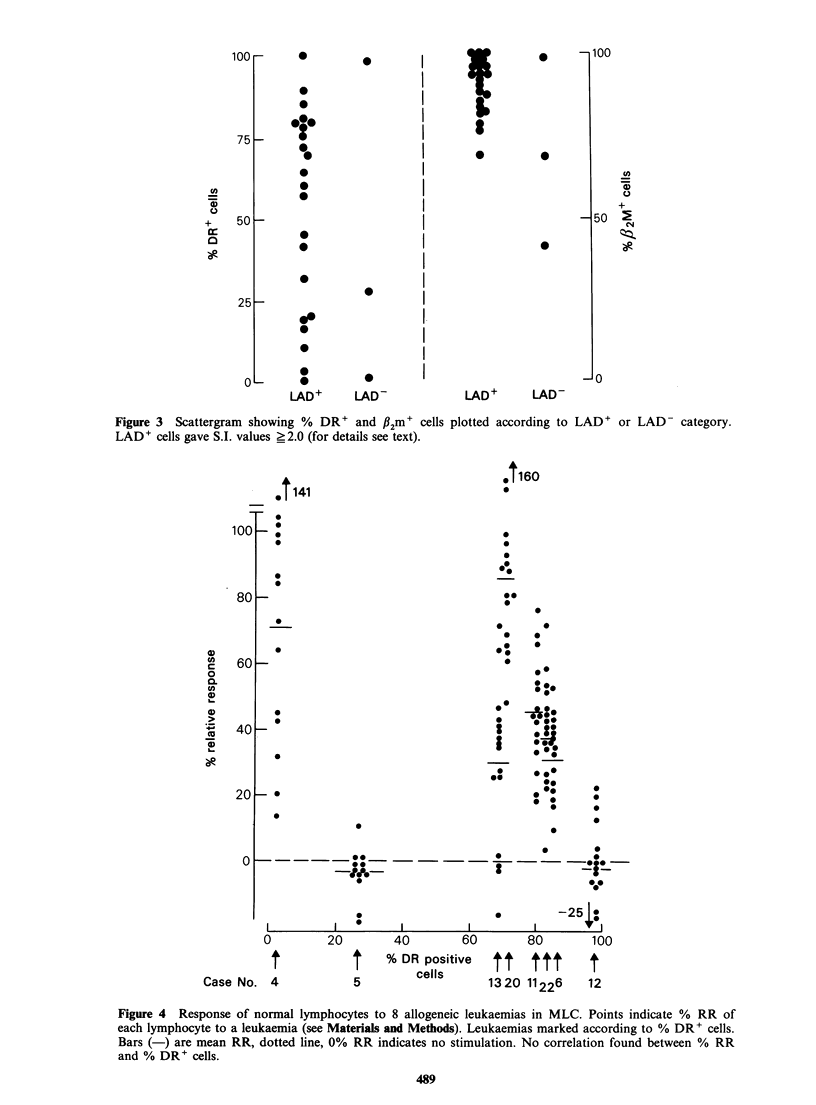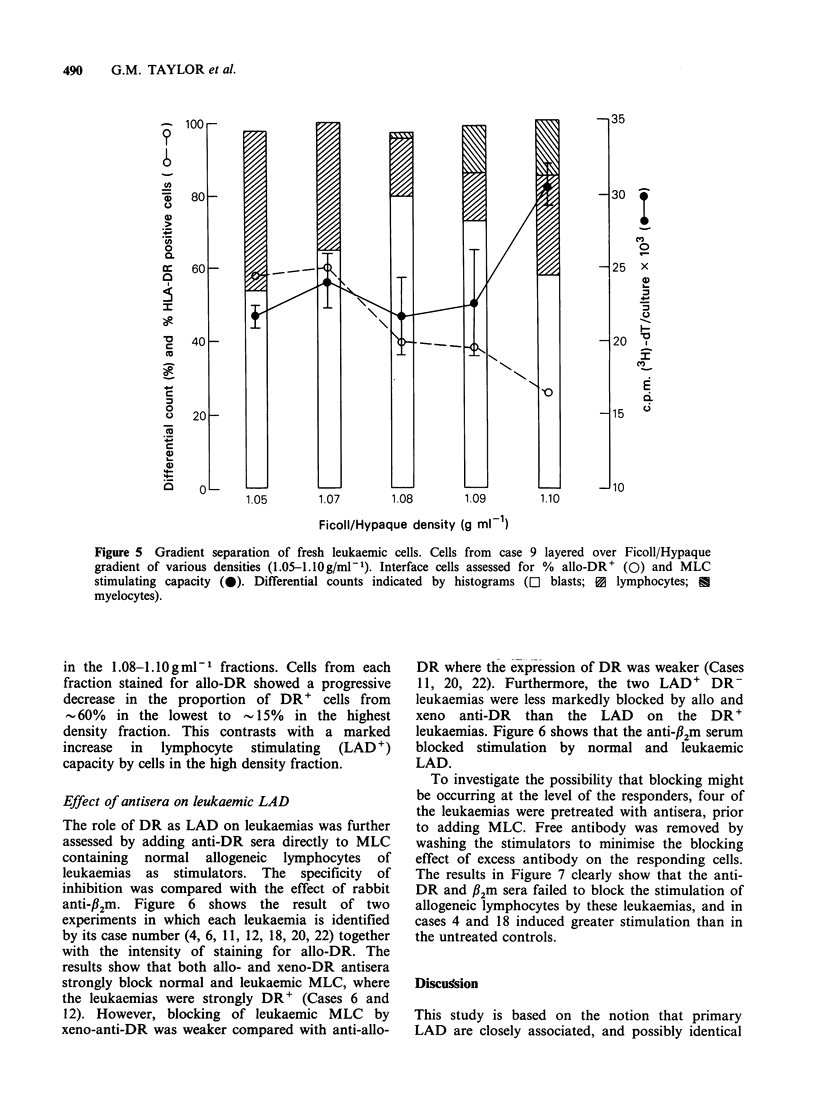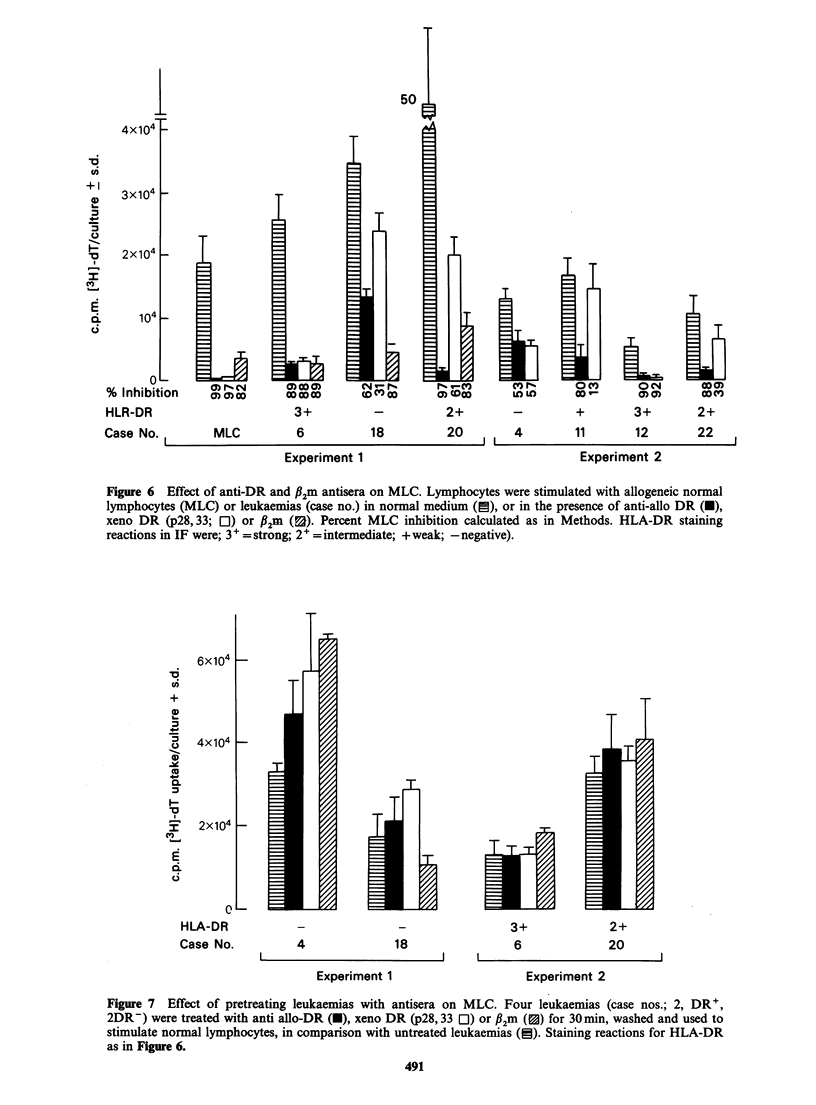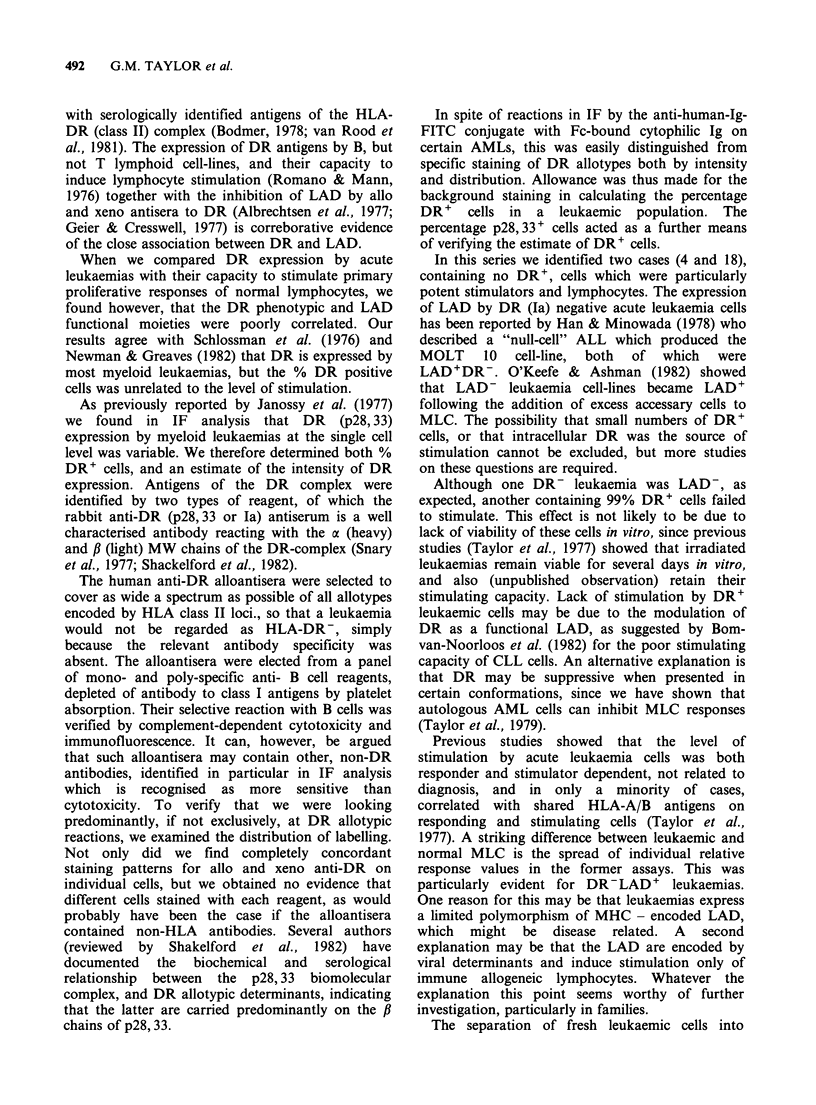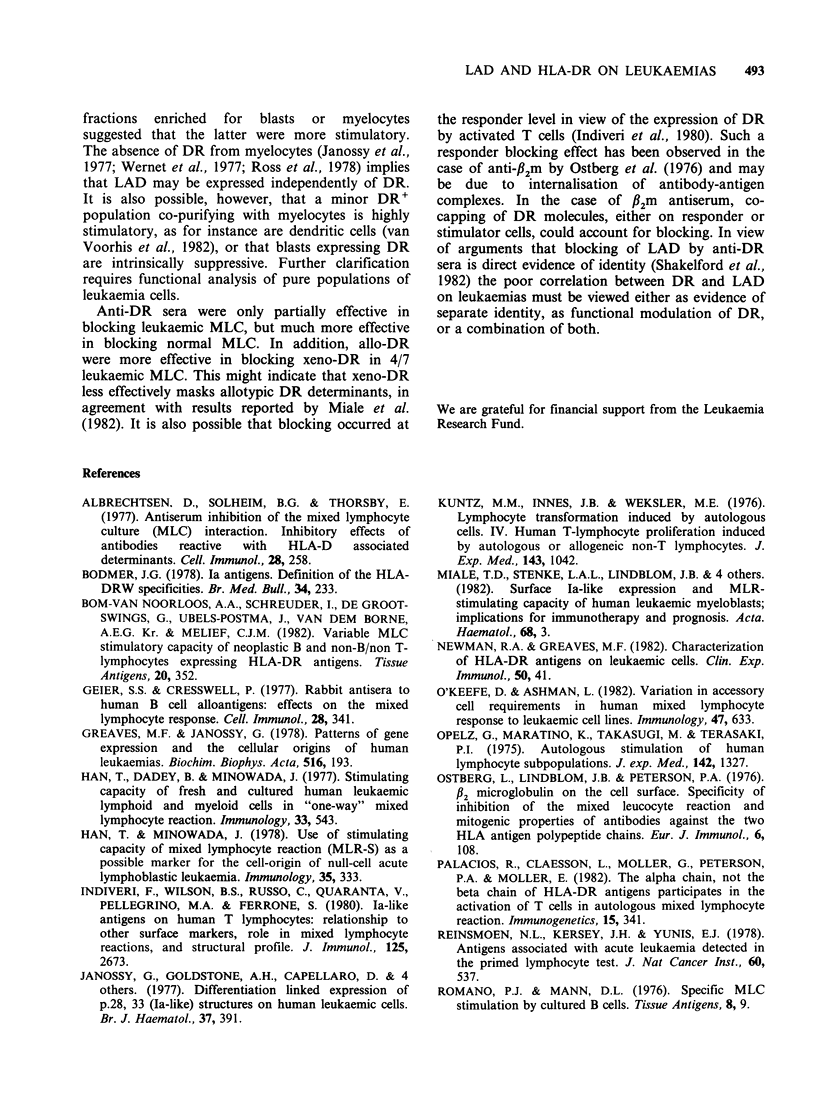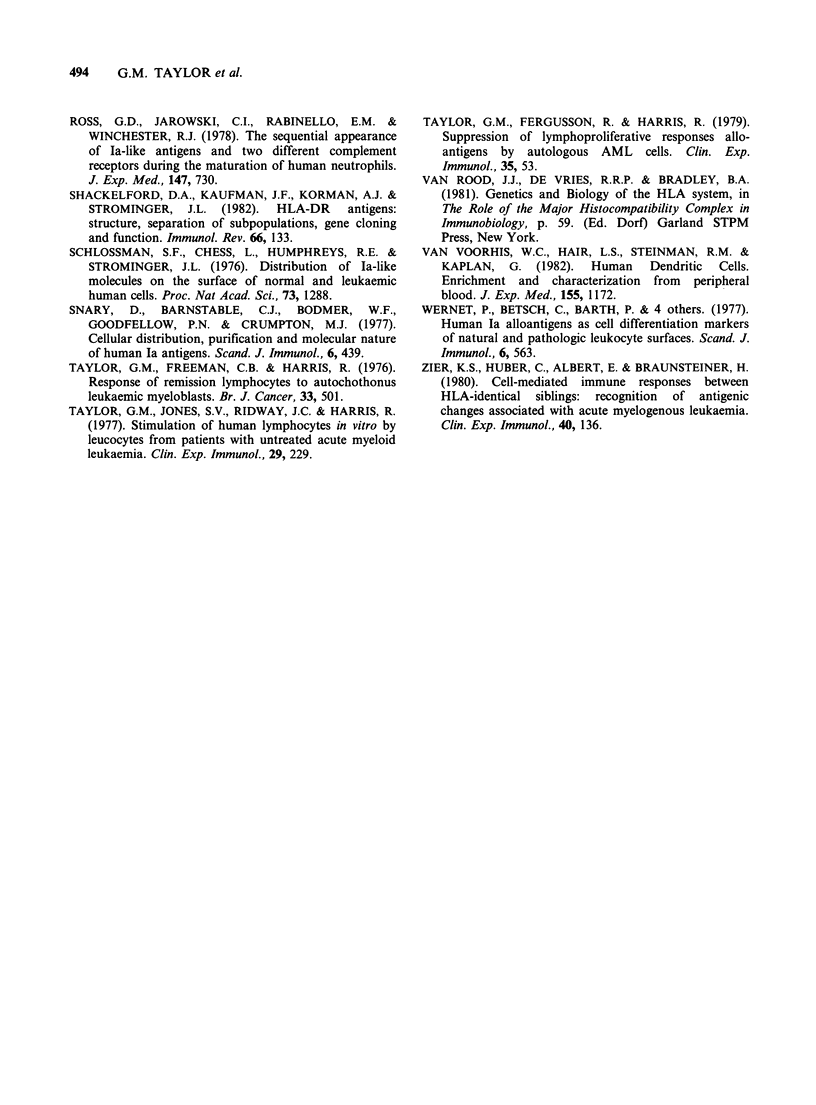# Lack of correlation between lymphocyte activating determinants and HLA-DR on acute leukaemias.

**DOI:** 10.1038/bjc.1984.76

**Published:** 1984-04

**Authors:** G. M. Taylor, J. C. Ridway, W. D. Fergusson, R. Harris

## Abstract

The expression of allogenic lymphocyte-activating determinants (LAD) on 25 acute leukaemias has been compared with the expression of cell-surface antigens identified by HLA-DR allo- and xeno-antisera. The close correlation between LAD and DR known to occur on normal lymphocytes was not found in leukaemias. Twenty-two LAD+ leukaemias included 2 DR- cases, whilst 2 LAD- leukaemias were DR+. With the exception of 3 leukaemias all were strongly beta 2 microglobulin+. No correlation was found between the % DR+ cells and the level of lymphocyte stimulation. Separation of leukaemia cells on Ficoll gradients into fractions containing different proportions of DR+ cells did not correlate with LAD expression. Furthermore, antisera to DR antigens only partially blocked leukaemic LAD. The results support the notion that LAD on acute leukaemias are not necessarily associated with or identical to HLA-DR antigens, and that the lymphocyte activating capacity of HLA-DR may be modulated.


*Br. J. Cancer* (1984), **49**, 485–494

### Lack of correlation between lymphocyte activating determinants and HLA-DR on acute leukaemias

G.M. Taylor[1], J.C. Ridway[2], W.D. Fergusson[1] & R. Harris[1]

[1]*Immunogenetics Laboratory, Department of Medical Genetics, St. Mary's Hospital, Manchester, M3 0JH,* [2]*Department of Haematology, Blackpool Victoria Hospital, Blackpool, UK.*

**Summary** The expression of allogenic lymphocyte-activating determinants (LAD) on 25 acute leukaemias has been compared with the expression of cell-surface antigens identified by HLA-DR allo- and xeno-antisera. The close correlation between LAD and DR known to occur on normal lymphocytes was not found in leukaemias. Twenty-two $LAD^+$ leukaemias included 2 $DR^-$ cases, whilst 2 $LAD^-$ leukaemias were $DR^+$. With the exception of 3 leukaemias all were strongly $\beta_2$ $microglobulin^+$. No correlation was found between the % $DR^+$ cells and the level of lymphocyte stimulation. Separation of leukaemia cells on Ficoll gradients into fractions containing different proportions of $DR^+$ cells did not correlate with LAD expression. Furthermore, antisera to DR antigens only partially blocked leukaemic LAD. The results support the notion that LAD on acute leukaemias are not necessarily associated with or identical to HLA-DR antigens, and that the lymphocyte activating capacity of HLA-DR may be modulated.

Lymphocyte-activating determinants (LAD) encoded by the HLA-D region of the human major histocompatibility complex (MHC) are responsible for stimulation of $^3$H-thymidine ([$^3$H]-dT) uptake in primary mixed lymphocyte culture (van Rood *et al.*, 1981). Acute leukaemias express lymphocyte-activating determinants which stimulate autologous and allogeneic lymphocytes (Taylor *et al.* 1976; 1977; Han *et al.*, 1977). Family studies indicate that LAD on leukaemic blasts may be encoded by the MHC, though stimulation of autologous and HLA-identical sib lymphocytes by these cells suggest the expression of other, possibly leukaemia-specific, LAD (Reinsmoen *et al.*, 1978; Zier *et al.*, 1980).

MHC-encoded LAD are closely associated, and perhaps identical with serologically defined antigens of the HLA-DR complex, expressed predominantly by resting B lymphocytes (Bodmer, 1978). Most acute myeloid leukaemias express cell-surface HLA-DR (Schlossman *et al.*, 1976; Janossy *et al.*, 1977; Newman & Greaves, 1982) which implies that such cells should be $LAD^+$. DR-associated LAD on leukaemias may also stimulate autologous lymphocytes, in the same way that the autologous MLC induced by B lymphocytes (Opelz *et al.*, 1975; Kuntz *et al.*, 1976) is under HLA-DR control (Palacios *et al.*, 1982). In this respect, leukaemic LAD may correspond functionally to LAD expressed by stem cells, in similar fashion to other differentiation related antigens on leukaemias (Greaves & Janossy, 1978) rather than to neodeterminants induced by malignant transformation.

Previous studies (Taylor *et al.*, 1977) showed that AML cells induce wide variations in lymphocyte stimulation, which do not seem to be related to the expression of DR-complex (Ia) antigens (Miale *et al.*, 1982). From similar observations in $DR^+$ chronic lymphocytic leukaemia (Bom-van-Noorloos *et al.*, 1982) it has been suggested that phenotypically expressed DR may be functionally modulated as LAD.

In order to establish whether DR expression is closely associated with LAD on acute leukaemias, we have studied 25 cases containing high numbers of blast cells, variable proportions of which are $DR^+$. We found that DR expression was not closely correlated with LAD, suggesting that DR independent LAD are expressed by some leukaemias, whilst on others, DR is functionally inactive as an LAD.

### Materials and methods

### *Cells*

Heparinised peripheral blood from 25 acute leukaemia patients, mostly with a high percentage of blasts (≧60%) was obtained before treatment. Diagnoses were made on bone marrow and peripheral blood smears, using standard cytological methods (PAS, peroxidase, Sudan black, esterase).

Correspondence: G.M. Taylor, Department of Medical Genetics, St Mary's Hospital, Hathersage Road, Manchester, M13 0JH.
Received 4 July 1983; accepted 4 January 1984.

© The Macmillan Press Ltd., 1984

Leucocytes were separated on lymphocyte separation medium (LSM, Flow Laboratories, Scotland) as previously described (Taylor *et al.*, 1979). The leucocytes were washed in Hanks Balanced Salt Solution containing 2% newborn calf serum (HBS-NCS) then resuspended in RPM1-1640 containing 10% foetal calf serum (1640-FCS) and 10% dimethyl sulphoxide (DMSO). Differential counts of leukaemias after $LN_2$ storage showed only minor changes in the proportion of cell types (data not shown).

Normal lymphocytes were prepared from peripheral blood by separation on LSM as above. Responding and stimulating cells in MLC were prepared in RPM1-1640 culture medium, containing a 10% heat-inactivated human AB serum (CM-AB).

*Mixed leucocyte cultures (MLC)*

Leukaemias and normal lymphocytes as stimulators in MLC were inactivated in a $^{137}$Cs-irradiator (6 K rad). Responders and stimulators at $5 \times 10^5 ml^{-1}$ in CM-AB were dispensed in quadruplicate into microplates with round wells (M24 ART, Sterilin, UK) as previously described (Taylor *et al.*, 1979). The microplates were incubated in humidified 95% air/5% $CO_2$ for 5 days, labelled overnight with 2 $\mu$Ci per well methyl-(3H)-thymidine ([$^3$H]-dT, sp. act. 20 Ci mmol$^{-1}$, Radiochemical Centre, Amersham, UK) and harvested onto glass-fibre filters using a Skatron/Titertek cell harvester (Flow Laboratories, Scotland). The filters were dried and counted in a toluene-based scintillant on a Beckman LS3155T liquid scintillation counter.

Blocking of MLC was determined by adding decomplemented antisera (see below – anti-DR, p28, 33 or $\beta_2$m) directly to normal or leukaemic MLCs on day 0, or by pretreating leukaemias with antisera, washing then adding them to responding lymphocytes.

Results of MLCs in counts min$^{-1}$ (cpm) [$^3$H]-dT uptake are expressed as:

1. cpm $\pm$ s.d.
2. Stimulation indices $(SI) = R \times S / R + S$, where $R \times S$ is the cpm for the responder (R) stimulated with the leukaemia(s) in MLC, and $R + S$, the cpm for responders and stimulators cultured alone. Leukaemias giving $SI \leqq 1.0$ were considered as LAD$^-$ and $\geqq 2.0$ as LAD$^+$.
3. % *Relative responses (RR)* =

$$\frac{(R \times S) - (R + S)}{\begin{matrix}(R \times \text{pooled} \\ \text{allogeneic} \\ \text{lymphocytes})\end{matrix} - \begin{matrix}(R \times \text{autologous} \\ \text{lymphocytes})\end{matrix}} \times 100$$

4. % *MLC inhibition* =

$$1 - \frac{(R \times S) \text{ Test antiserum}}{(R \times S) \text{ normal serum}} \times 100$$

*Antisera*

Rabbit antiserum to the p28, 33 bimolecular complex was a gift from Dr M.J. Crumpton (ICRF, London, UK). In this paper it is referred to as xeno-anti-DR serum, although such antisera recognise $\alpha$ and $\beta$ chains encoded by other class II loci (Shackelford *et al.*, 1982). Rabbit anti-$\beta_2$m was obtained from Dakopatts (Copenhagen, Denmark).

Alloantisera were obtained from multiparous females, kidney transplant recipients and leukaemic patients immunized with allogeneic leukaemia cells, and rendered B-cell specific by absorption with packed, pooled blood platelets. These antisera were selected on the basis of positive complement dependent cytotoxic reactions on B-cells and negative reactions on T cells $(n = 30)$. The B-cell specificity was further tested on normal lymphocytes by indirect membrane immunofluorescence (IF, see below), combined with sheep erythrocyte (E) rosetting. Briefly, lymphocytes treated with alloantiserum and FITC-conjugated anti-human Ig were rosetted with neuraminidase-treated E overnight at 4° and examined for evidence of IF staining of non-rosetted (i.e. non-T) cells. Antisera giving such reactions were selected, whilst antisera reacting with rosetted (i.e. T) cells were rejected. Of the nine alloantisera selected, 2 were operationally monospecific for HLA-DR1 and DR5 in C-dependent cytotoxicity assays. However, since the objective was to identify most if not all DR specificities, the sera were combined to give three pools of three antisera. When further tested in membrane IF, these antisera reacted with all normal B cells, CLL cells, and B lymphoblastoid cell lines, but not with the T cell lines Molt-4, or CCRF-CEM. They are referred to as allo-anti-DR sera throughout this paper though they almost certainly contained antibodies to other HLA class II allotypic determinants.

*Immunofluorescence (IF) analysis*

Viable leucocytes ($10^6$ 100 $\mu l^{-1}$) were treated at 4° with 50–100 $\mu$l of xeno (p28, 33) (1/10) or polyspecific allo-anti DR (neat or 1/2) or anti-$\beta_2$m (1/10–1/20) for 30 min at 4°, washed PBS and stained with FITC-conjugated sheep anti-human Ig (for DR antisera) or FITC sheep anti-rabbit Ig (for p28, 33 and $\beta_2$m) (Wellcome Laboratories, Beckenham, UK). Control leucocytes were treated with normal human or rabbit serum, and stained as above. Cells were finally washed three times, resuspended in 50% glycerol in PBS and examined under a Zeiss Universal epifluorescence microscope.

A minimum of 200 leucocytes in each preparation were counted and the % positively stained cells calculated. The % positive cells in the control (normal sera treated) preparations were subtracted in each instance, to give a corrected percentage value. Cells were also scored for staining intensity on a 0–3 scale (0 = negative, $1^+$, $2^+$, and $3^+$ were respectively weak, intermediate and strong positive). In a minority of cases where the number of cells with Fc-bound human Ig was $\geq 10\%$, the difference in staining intensity between the allo-anti DR-sera compared with the control was used to assess whether DR was expressed. Since Fc receptors and DR were co-expressed on these cells, the % positively stained cells was computed on the basis of staining intensity. Each cell was tested with the three pooled antisera, and the pool giving the highest percentage in each case taken as the % allo-DR positive cells.

Double fluorochrome labelling of allo- and xeno-DR antigens was performed by incubating leucocytes sequentially with allo- and xeno- anti-DR sera then staining with a mixture of equal parts of 1/5 diluted FITC-F$(Ab)_2$ goat anti-human Ig and TRTC-F$(Ab)_2$ goat anti-rabbit Ig (heavy and light chain specific) antisera (Cappel Laboratories, Cockranville, USA). The cells were examined under a Leitz Dialux epifluorescence microscope using TRITC and FITC filters and a 50 times water-immersion objective. Only surface Ig negative leukaemias were used in the analysis.

### Results

### *HLA-DR expression by acute leukaemias*

We studied 25 acute leukaemias, selected because most contained $\geq 60\%$ blasts. Diagnostically they included 11 AML (cases 1–11), 2 undifferentiated AML (cases 12 and 13), one atypical AML (case 14), 6 AML (cases 15–20), 2 AMOL (cases 21 and 22), 2 ALL (cases 23 and 24) and one CML in blast crisis (case 25).

The percentage of cells expressing allo-DR was compared with xeno-DR (p28, 33) in membrane IF analysis, and the results plotted in Figure 1. In general the percentage of cells positive for both antigens were similar, except cases 11, 16, 9, 10, 23 and 25. Nonetheless, there was a significant correlation between the percentage of allo- and xeno-$DR^+$ cells ($r = 0.88$, $P < 0.01$).

However, to establish the steric relationship between allo and xeno DR antigens cells from 6 cases were double fluorochrome labelled. Allo-DR was detected by an FITC anti-human Ig and xeno-DR by a TRITC anti-rabbit Ig. Leukaemias were treated either with allo-DR or xeno-DR antisera alone or with both antisera sequentially, then with a mixture of the FITC and TRITC conjugates. Cells treated with one anti-DR serum only, reacted specifically with the relevant conjugate (i.e. FITC stained only allo-DR treated cells, TRITC stained only xeno-DR treated cells). In sequentially antiserum treated leukaemias, however, cells which were allo-$DR^+$ were also xeno-$DR^+$. At the single cell level, FITC and TRITC staining patterns completely overlapped.

No correlation was found between the percentage allo-$DR^+$ cells and the percentage blasts ($r = 0.43$, $P > 0.05$), as seen in Figure 2, indicating mixed $DR^+$ and $DR^-$ populations of blasts in most leukaemias. Only three leukaemias (cases 1, 2 and 5) contained $<75\%$ of $\beta_2m^+$ cells; the remaining cases contained strongly $\beta_2m^+$ blasts.

### *Expression of LAD on leukaemias*

Figure 3 shows the leukaemias plotted according to the expression of LAD in relation to the percentage of $DR^+$ and $\beta_2m^+$ cells. It is clear that cells classified as $LAD^+$ (see **Materials and methods**) contained quite different proportions of $DR^+$ cells; in particular two $LAD^+$ leukaemias (Cases 4 and 18) were $DR^-$. In addition, only one of the three $LAD^-$ leukaemias was also $DR^-$. A frequency plot (not shown) indicates a bimodal distribution of $LAD^+$ leukaemias in groups with $<20\%$ and $>50\%$ $DR^+$ blasts.

In Figure 4 the relationship between the level of stimulation (% RR) by 8 cases tested on 14–16 responders, and DR expression is shown. The wide distribution on RR values agrees with previous results (Taylor *et al.*, 1977) and bears little relationship to the percentage $DR^+$ cells. Two cases (4 and 12) containing 0 and 99% $DR^+$ cells were respectively $LAD^+$ and $LAD^-$. In addition three leukaemias (5, 13 and 12) induced negative RR values in a number of tests, perhaps indicating the induction of suppression. No correlation was found between mean RR and the % $DR^+$ cells or % blasts ($r = 0.21$ and $r = 0.05$ respectively, $P > 0.1$).

### *LAD on fractionated leukaemia cells*

Freshly obtained AML cells (Case 9) were layered over different densities of Ficoll/Hypaque (1.05–1.10 g ml$^{-1}$) in separate tubes, centrifuged for 30 min at 300 *g*, and the fractions analysed for LAD and DR. Figure 5 shows the main cell types in each fraction, with blasts predominating, particularly in the 1.08 g ml$^{-1}$ fraction. Lymphocytes were found in the lighter density fractions, but myelocytes formed an increasing proportion of cells collected

**Figure 1** Correlation between % allo-DR$^+$ and xeno-DR (p28, 33)$^+$ cells in 25 acute leukaemias, analysed by membrane immunofluorescence. Numbers refer to individual cases (see text). Correlation coefficient (r) = 0.8 ($P$ < 0.01). Linear regression analysis gave slope (—) with 95% confidence limits (---) showing close relationship between both types of cell.

**Figure 2** Percentage allo-DR$^+$ cells plotted against % blasts. Cases indicated by numbers (see **Figure 1** and text). Correlation coefficient (r) = 0.43 $P$ > 0.05. Regression analysis gave slope (—), no confidence limits.

**Figure 3** Scattergram showing % $DR^+$ and $\beta_2m^+$ cells plotted according to $LAD^+$ or $LAD^-$ category. $LAD^+$ cells gave S.I. values $\geq 2.0$ (for details see text).

**Figure 4** Response of normal lymphocytes to 8 allogeneic leukaemias in MLC. Points indicate % RR of each lymphocyte to a leukaemia (see **Materials and Methods**). Leukaemias marked according to % $DR^+$ cells. Bars (—) are mean RR, dotted line, 0% RR indicates no stimulation. No correlation found between % RR and % $DR^+$ cells.

**Figure 5** Gradient separation of fresh leukaemic cells. Cells from case 9 layered over Ficoll/Hypaque gradient of various densities (1.05–1.10 g/ml$^{-1}$). Interface cells assessed for % allo-DR$^+$ (○) and MLC stimulating capacity (●). Differential counts indicated by histograms (□ blasts; ▨ lymphocytes; ▧ myelocytes).

in the 1.08–1.10 g ml$^{-1}$ fractions. Cells from each fraction stained for allo-DR showed a progressive decrease in the proportion of DR$^+$ cells from ~60% in the lowest to ~15% in the highest density fraction. This contrasts with a marked increase in lymphocyte stimulating (LAD$^+$) capacity by cells in the high density fraction.

*Effect of antisera on leukaemic LAD*

The role of DR as LAD on leukaemias was further assessed by adding anti-DR sera directly to MLC containing normal allogeneic lymphocytes of leukaemias as stimulators. The specificity of inhibition was compared with the effect of rabbit anti-$\beta_2$m. Figure 6 shows the result of two experiments in which each leukaemia is identified by its case number (4, 6, 11, 12, 18, 20, 22) together with the intensity of staining for allo-DR. The results show that both allo- and xeno-DR antisera strongly block normal and leukaemic MLC, where the leukaemias were strongly DR$^+$ (Cases 6 and 12). However, blocking of leukaemic MLC by xeno-anti-DR was weaker compared with anti-allo-DR where the expression of DR was weaker (Cases 11, 20, 22). Furthermore, the two LAD$^+$ DR$^-$ leukaemias were less markedly blocked by allo and xeno anti-DR than the LAD on the DR$^+$ leukaemias. Figure 6 shows that the anti-$\beta_2$m serum blocked stimulation by normal and leukaemic LAD.

To investigate the possibility that blocking might be occurring at the level of the responders, four of the leukaemias were pretreated with antisera, prior to adding MLC. Free antibody was removed by washing the stimulators to minimise the blocking effect of excess antibody on the responding cells. The results in Figure 7 clearly show that the anti-DR and $\beta_2$m sera failed to block the stimulation of allogeneic lymphocytes by these leukaemias, and in cases 4 and 18 induced greater stimulation than in the untreated controls.

### Discussion

This study is based on the notion that primary LAD are closely associated, and possibly identical

**Figure 6** Effect of anti-DR and $\beta_2$m antisera on MLC. Lymphocytes were stimulated with allogeneic normal lymphocytes (MLC) or leukaemias (case no.) in normal medium (▤), or in the presence of anti-allo DR (■), xeno DR (p28, 33; □) or $\beta_2$m (▨). Percent MLC inhibition calculated as in Methods. HLA-DR staining reactions in IF were; $3^+$ = strong; $2^+$ = intermediate; +weak; −negative).

**Figure 7** Effect of pretreating leukaemias with antisera on MLC. Four leukaemias (case nos.; 2, $DR^+$, $2DR^-$) were treated with anti allo-DR (■), xeno DR (p28, 33 □) or $\beta_2$m (▨) for 30 min, washed and used to stimulate normal lymphocytes, in comparison with untreated leukaemias (▤). Staining reactions for HLA-DR as in **Figure 6**.

with serologically identified antigens of the HLA-DR (class II) complex (Bodmer, 1978; van Rood *et al.*, 1981). The expression of DR antigens by B, but not T lymphoid cell-lines, and their capacity to induce lymphocyte stimulation (Romano & Mann, 1976) together with the inhibition of LAD by allo and xeno antisera to DR (Albrechtsen *et al.*, 1977; Geier & Cresswell, 1977) is correborative evidence of the close association between DR and LAD.

When we compared DR expression by acute leukaemias with their capacity to stimulate primary proliferative responses of normal lymphocytes, we found however, that the DR phenotypic and LAD functional moieties were poorly correlated. Our results agree with Schlossman *et al.* (1976) and Newman & Greaves (1982) that DR is expressed by most myeloid leukaemias, but the % DR positive cells was unrelated to the level of stimulation.

As previously reported by Janossy *et al.* (1977) we found in IF analysis that DR (p28, 33) expression by myeloid leukaemias at the single cell level was variable. We therefore determined both % $DR^+$ cells, and an estimate of the intensity of DR expression. Antigens of the DR complex were identified by two types of reagent, of which the rabbit anti-DR (p28, 33 or Ia) antiserum is a well characterised antibody reacting with the $\alpha$ (heavy) and $\beta$ (light) MW chains of the DR-complex (Snary *et al.*, 1977; Shackelford *et al.*, 1982).

The human anti-DR alloantisera were selected to cover as wide a spectrum as possible of all allotypes encoded by HLA class II loci., so that a leukaemia would not be regarded as HLA-$DR^-$, simply because the relevant antibody specificity was absent. The alloantisera were elected from a panel of mono- and poly-specific anti- B cell reagents, depleted of antibody to class I antigens by platelet absorption. Their selective reaction with B cells was verified by complement-dependent cytotoxicity and immunofluorescence. It can, however, be argued that such alloantisera may contain other, non-DR antibodies, identified in particular in IF analysis which is recognised as more sensitive than cytotoxicity. To verify that we were looking predominantly, if not exclusively, at DR allotypic reactions, we examined the distribution of labelling. Not only did we find completely concordant staining patterns for allo and xeno anti-DR on individual cells, but we obtained no evidence that different cells stained with each reagent, as would probably have been the case if the alloantisera contained non-HLA antibodies. Several authors (reviewed by Shakelford *et al.*, 1982) have documented the biochemical and serological relationship between the p28, 33 biomolecular complex, and DR allotypic determinants, indicating that the latter are carried predominantly on the $\beta$ chains of p28, 33.

In spite of reactions in IF by the anti-human-Ig-FITC conjugate with Fc-bound cytophilic Ig on certain AMLs, this was easily distinguished from specific staining of DR allotypes both by intensity and distribution. Allowance was thus made for the background staining in calculating the percentage $DR^+$ cells in a leukaemic population. The percentage p28, $33^+$ cells acted as a further means of verifying the estimate of $DR^+$ cells.

In this series we identified two cases (4 and 18), containing no $DR^+$, cells which were particularly potent stimulators and lymphocytes. The expression of LAD by DR (Ia) negative acute leukaemia cells has been reported by Han & Minowada (1978) who described a "null-cell" ALL which produced the MOLT 10 cell-line, both of which were $LAD^+DR^-$. O'Keefe & Ashman (1982) showed that $LAD^-$ leukaemia cell-lines became $LAD^+$ following the addition of excess accessary cells to MLC. The possibility that small numbers of $DR^+$ cells, or that intracellular DR was the source of stimulation cannot be excluded, but more studies on these questions are required.

Although one $DR^-$ leukaemia was $LAD^-$, as expected, another containing 99% $DR^+$ cells failed to stimulate. This effect is not likely to be due to lack of viability of these cells *in vitro*, since previous studies (Taylor *et al.*, 1977) showed that irradiated leukaemias remain viable for several days *in vitro*, and also (unpublished observation) retain their stimulating capacity. Lack of stimulation by $DR^+$ leukaemic cells may be due to the modulation of DR as a functional LAD, as suggested by Bom-van-Noorloos *et al.* (1982) for the poor stimulating capacity of CLL cells. An alternative explanation is that DR may be suppressive when presented in certain conformations, since we have shown that autologous AML cells can inhibit MLC responses (Taylor *et al.*, 1979).

Previous studies showed that the level of stimulation by acute leukaemia cells was both responder and stimulator dependent, not related to diagnosis, and in only a minority of cases, correlated with shared HLA-A/B antigens on responding and stimulating cells (Taylor *et al.*, 1977). A striking difference between leukaemic and normal MLC is the spread of individual relative response values in the former assays. This was particularly evident for $DR^-LAD^+$ leukaemias. One reason for this may be that leukaemias express a limited polymorphism of MHC – encoded LAD, which might be disease related. A second explanation may be that the LAD are encoded by viral determinants and induce stimulation only of immune allogeneic lymphocytes. Whatever the explanation this point seems worthy of further investigation, particularly in families.

The separation of fresh leukaemic cells into fractions enriched for blasts or myelocytes suggested that the latter were more stimulatory. The absence of DR from myelocytes (Janossy *et al.*, 1977; Wernet *et al.*, 1977; Ross *et al.*, 1978) implies that LAD may be expressed independently of DR. It is also possible, however, that a minor DR$^+$ population co-purifying with myelocytes is highly stimulatory, as for instance are dendritic cells (van Voorhis *et al.*, 1982), or that blasts expressing DR are intrinsically suppressive. Further clarification requires functional analysis of pure populations of leukaemia cells.

Anti-DR sera were only partially effective in blocking leukaemic MLC, but much more effective in blocking normal MLC. In addition, allo-DR were more effective in blocking xeno-DR in 4/7 leukaemic MLC. This might indicate that xeno-DR less effectively masks allotypic DR determinants, in agreement with results reported by Miale *et al.* (1982). It is also possible that blocking occurred at the responder level in view of the expression of DR by activated T cells (Indiveri *et al.*, 1980). Such a responder blocking effect has been observed in the case of anti-$\beta_2$m by Ostberg *et al.* (1976) and may be due to internalisation of antibody-antigen complexes. In the case of $\beta_2$m antiserum, co-capping of DR molecules, either on responder or stimulator cells, could account for blocking. In view of arguments that blocking of LAD by anti-DR sera is direct evidence of identity (Shakelford *et al.*, 1982) the poor correlation between DR and LAD on leukaemias must be viewed either as evidence of separate identity, as functional modulation of DR, or a combination of both.

We are grateful for financial support from the Leukaemia Research Fund.

### References

ALBRECHTSEN, D., SOLHEIM, B.G. & THORSBY, E. (1977). Antiserum inhibition of the mixed lymphocyte culture (MLC) interaction. Inhibitory effects of antibodies reactive with HLA-D associated determinants. *Cell. Immunol.*, **28**, 258.

BODMER, J.G. (1978). Ia antigens. Definition of the HLA-DRW specificities. *Br. Med. Bull.*, **34**, 233.

BOM-VAN NOORLOOS, A.A., SCHREUDER, I., DE GROOT-SWINGS, G., UBELS-POSTMA, J., VAN DEM BORNE, A.E.G. Kr. & MELIEF, C.J.M. (1982). Variable MLC stimulatory capacity of neoplastic B and non-B/non T-lymphocytes expressing HLA-DR antigens. *Tissue Antigens*, **20**, 352.

GEIER, S.S. & CRESSWELL, P. (1977). Rabbit antisera to human B cell alloantigens: effects on the mixed lymphocyte response. *Cell. Immunol.*, **28**, 341.

GREAVES, M.F. & JANOSSY, G. (1978). Patterns of gene expression and the cellular origins of human leukaemias. *Biochim. Biophys. Acta*, **516**, 193.

HAN, T., DADEY, B. & MINOWADA, J. (1977). Stimulating capacity of fresh and cultured human leukaemic lymphoid and myeloid cells in "one-way" mixed lymphocyte reaction. *Immunology*, **33**, 543.

HAN, T. & MINOWADA, J. (1978). Use of stimulating capacity of mixed lymphocyte reaction (MLR-S) as a possible marker for the cell-origin of null-cell acute lymphoblastic leukaemia. *Immunology*, **35**, 333.

INDIVERI, F., WILSON, B.S., RUSSO, C., QUARANTA, V., PELLEGRINO, M.A. & FERRONE, S. (1980). Ia-like antigens on human T lymphocytes: relationship to other surface markers, role in mixed lymphocyte reactions, and structural profile. *J. Immunol.*, **125**, 2673.

JANOSSY, G., GOLDSTONE, A.H., CAPELLARO, D. & 4 others. (1977). Differentiation linked expression of p.28, 33 (Ia-like) structures on human leukaemic cells. *Br. J. Haematol.*, **37**, 391.

KUNTZ, M.M., INNES, J.B. & WEKSLER, M.E. (1976). Lymphocyte transformation induced by autologous cells. IV. Human T-lymphocyte proliferation induced by autologous or allogeneic non-T lymphocytes. *J. Exp. Med.*, **143**, 1042.

MIALE, T.D., STENKE, L.A.L., LINDBLOM, J.B. & 4 others. (1982). Surface Ia-like expression and MLR-stimulating capacity of human leukaemic myeloblasts; implications for immunotherapy and prognosis. *Acta. Haematol.*, **68**, 3.

NEWMAN, R.A. & GREAVES, M.F. (1982). Characterization of HLA-DR antigens on leukaemic cells. *Clin. Exp. Immunol.*, **50**, 41.

O'KEEFE, D. & ASHMAN, L. (1982). Variation in accessory cell requirements in human mixed lymphocyte response to leukaemic cell lines. *Immunology*, **47**, 633.

OPELZ, G., MARATINO, K., TAKASUGI, M. & TERASAKI, P.I. (1975). Autologous stimulation of human lymphocyte subpopulations. *J. exp. Med.*, **142**, 1327.

OSTBERG, L., LINDBLOM, J.B. & PETERSON, P.A. (1976). $\beta_2$ microglobulin on the cell surface. Specificity of inhibition of the mixed leucocyte reaction and mitogenic properties of antibodies against the two HLA antigen polypeptide chains. *Eur. J. Immunol.*, **6**, 108.

PALACIOS, R., CLAESSON, L., MOLLER, G., PETERSON, P.A. & MOLLER, E. (1982). The alpha chain, not the beta chain of HLA-DR antigens participates in the activation of T cells in autologous mixed lymphocyte reaction. *Immunogenetics*, **15**, 341.

REINSMOEN, N.L., KERSEY, J.H. & YUNIS, E.J. (1978). Antigens associated with acute leukaemia detected in the primed lymphocyte test. *J. Nat Cancer Inst.*, **60**, 537.

ROMANO, P.J. & MANN, D.L. (1976). Specific MLC stimulation by cultured B cells. *Tissue Antigens*, **8**, 9.

ROSS, G.D., JAROWSKI, C.I., RABINELLO, E.M. & WINCHESTER, R.J. (1978). The sequential appearance of Ia-like antigens and two different complement receptors during the maturation of human neutrophils. *J. Exp. Med.*, **147**, 730.

SHACKELFORD, D.A., KAUFMAN, J.F., KORMAN, A.J. & STROMINGER, J.L. (1982). HLA-DR antigens: structure, separation of subpopulations, gene cloning and function. *Immunol. Rev.* **66**, 133.

SCHLOSSMAN, S.F., CHESS, L., HUMPHREYS, R.E. & STROMINGER, J.L. (1976). Distribution of Ia-like molecules on the surface of normal and leukaemic human cells. *Proc. Nat Acad. Sci.*, **73**, 1288.

SNARY, D., BARNSTABLE, C.J., BODMER, W.F., GOODFELLOW, P.N. & CRUMPTON, M.J. (1977). Cellular distribution, purification and molecular nature of human Ia antigens. *Scand. J. Immunol.*, **6**, 439.

TAYLOR, G.M., FREEMAN, C.B. & HARRIS, R. (1976). Response of remission lymphocytes to autochothonus leukaemic myeloblasts. *Br. J. Cancer*, **33**, 501.

TAYLOR, G.M., JONES, S.V., RIDWAY, J.C. & HARRIS, R. (1977). Stimulation of human lymphocytes *in vitro* by leucocytes from patients with untreated acute myeloid leukaemia. *Clin. Exp. Immunol.*, **29**, 229.

TAYLOR, G.M., FERGUSSON, R. & HARRIS, R. (1979). Suppression of lymphoproliferative responses alloantigens by autologous AML cells. *Clin. Exp. Immunol.*, **35**, 53.

VAN ROOD, J.J., DE VRIES, R.R.P. & BRADLEY, B.A. (1981). Genetics and Biology of the HLA system, in *The Role of the Major Histocompatibility Complex in Immunobiology*, p. 59. (Ed. Dorf) Garland STPM Press, New York.

VAN VOORHIS, W.C., HAIR, L.S., STEINMAN, R.M. & KAPLAN, G. (1982). Human Dendritic Cells. Enrichment and characterization from peripheral blood. *J. Exp. Med.*, **155**, 1172.

WERNET, P., BETSCH, C., BARTH, P. & 4 others. (1977). Human Ia alloantigens as cell differentiation markers of natural and pathologic leukocyte surfaces. *Scand. J. Immunol.*, **6**, 563.

ZIER, K.S., HUBER, C., ALBERT, E. & BRAUNSTEINER, H. (1980). Cell-mediated immune responses between HLA-identical siblings: recognition of antigenic changes associated with acute myelogenous leukaemia. *Clin. Exp. Immunol.*, **40**, 136.